# Inflammatory biomarkers and physiomarkers of late-onset sepsis and necrotizing enterocolitis in premature infants

**DOI:** 10.3389/fped.2024.1337849

**Published:** 2024-01-19

**Authors:** Rupin Kumar, Sherry L. Kausch, Angela K. S. Gummadi, Karen D. Fairchild, Mayuresh M. Abhyankar, William A. Petri, Brynne A. Sullivan

**Affiliations:** ^1^ Department of Pediatrics, Division of Neonatology, University of Kentucky College of Medicine, Lexington, KY, United States; ^2^ Department of Pediatrics, Division of Neonatology, University of Virginia School of Medicine, Charlottesville, VA, United States; ^3^ Department of Internal Medicine, Division of Infectious Diseases, University of Virginia School of Medicine, Charlottesville, VA, United States

**Keywords:** prematurity, neonatal sepsis, biomarkers, inflammation, heart rate, oxygenation, cardiorespiratory

## Abstract

**Background:**

Early diagnosis of late-onset sepsis (LOS) and necrotizing enterocolitis (NEC) in very low birth weight (VLBW, <1,500 g) infants is challenging due to non-specific clinical signs. Inflammatory biomarkers increase in response to infection, but non-infectious conditions also cause inflammation. Cardiorespiratory data contain physiological biomarkers, or physiomarkers, of sepsis that may be useful in combination with inflammatory hematologic biomarkers for sepsis diagnosis.

**Objectives:**

To determine whether inflammatory biomarkers measured at the time of LOS or NEC diagnosis differ from times without infection and whether biomarkers correlate with cardiorespiratory sepsis physiomarkers in VLBW infants.

**Methods:**

Remnant plasma sample collection from VLBW infants occurred with blood draws for routine laboratory testing and suspected sepsis. We analyzed 11 inflammatory biomarkers and a pulse oximetry sepsis warning score (POWS). We compared biomarker levels obtained at the time of gram-negative (GN) bacteremia or NEC, gram-positive (GP) bacteremia, negative blood cultures, and no suspected infection.

**Results:**

We analyzed 188 samples in 54 VLBW infants. Several biomarkers were increased at the time of GN LOS or NEC diagnosis compared with all other samples. POWS was higher in patients with LOS and correlated with five biomarkers. IL-6 had 78% specificity at 100% sensitivity to detect GN LOS or NEC and added information to POWS.

**Conclusion(s):**

Inflammatory plasma biomarkers discriminate sepsis due to GN bacteremia or NEC and correlate with cardiorespiratory physiomarkers.

## 1 Introduction

Sepsis and necrotizing enterocolitis (NEC) lead to significant morbidity and mortality in premature infants (1). Inflammation due to sepsis and NEC can cause end-organ damage (2, 3), including brain injury, which leads to long-term impairment (4–6). Early recognition and treatment may improve outcomes, but signs and symptoms often overlap with non-infectious conditions (7). Therefore, a diagnosis is often made once the infection is advanced, and on the other hand, empiric antibiotics are given when there is no infection (8). Clinicians have limited tests and data to guide decisions on the likelihood of infection.

Inflammatory molecules, upregulated as part of the immune response to infection, hold promise as biomarkers of neonatal sepsis. A biomarker with sufficient diagnostic accuracy could be a useful screening tool to aid in deciding whether to start antibiotics or continue monitoring for further signs of sepsis. Many studies have evaluated biomarkers for neonatal sepsis in premature infants (9–12). But, few have been translated into clinical care (13). Decisions to start or stop antibiotics in the NICU largely rely on the clinician's assessment of clinical signs and non-specific laboratory tests, such as C-reactive protein (CRP) (14) and complete blood count (CBC) components (15).

Despite extensive research, no single blood, stool, or urine biomarker has been adopted in clinical practice for its predictive accuracy to diagnose neonatal sepsis and start antibiotic therapy (16). Physiologic sepsis biomarkers, such as abnormal vital sign patterns, can be detected using analysis of continuous vital sign data from standard bedside monitors. We previously used predictive analytics and a multicenter cohort of very low birth weight (VLBW, <1,500 g) preterm infants to develop and externally validate a cardiorespiratory sepsis risk model (17). The model, called Pulse Oximetry Warning Score (POWS), detects physiologic biomarkers of abnormal heart rate and SpO2 patterns that occur near the clinical diagnosis of late-onset sepsis and calculates the relative risk of blood culture-positive sepsis in the subsequent 24 h (17). POWS has yet to be implemented in clinical practice, so we sought to understand how inflammatory biomarkers will correlate and add to cardiorespiratory markers of sepsis. Combining information from hematologic and physiologic inflammatory biomarkers could improve the accuracy of sepsis diagnosis over using either alone.

While many prior studies have analyzed inflammatory biomarkers in premature infants at the time of suspected sepsis (10, 18–20), few have analyzed baseline levels or changes over time in patients who develop sepsis (21, 22). Inflammatory biomarkers may be less useful in premature infants with non-infectious conditions that lead to inflammation, including respiratory distress syndrome and chronic lung disease. To understand the potential utility of biomarkers in VLBW infants, more data are needed. In this study, we aim to identify inflammatory biomarkers that discriminate sepsis from baseline levels and non-sepsis conditions in VLBW infants. To do this, we measured multiple biomarkers in samples collected from remnant plasma, weekly when available and at the time of evaluation for late-onset sepsis or NEC.

## 2 Methods

### 2.1 Study design

This was a prospective, observational cohort study conducted at a single center Level IV academic NICU. The Institutional Review Board approved this study with consent from a parent (IRB #HSR20902). We prospectively enrolled VLBW infants within seven days of birth and collected clinical data and remnant plasma from blood drawn for suspected sepsis and for routine laboratory testing. Clinical data and culture results were recorded from the electronic health record. We classified samples according to the indication for the laboratory tests ordered and the final diagnosis of a workup for LOS or NEC (see “clinical definitions” section below).

### 2.2 Sample collection

The study protocol did not require any extra blood draws and did not influence the clinical team's decisions on ordering blood tests. Nurses collected blood samples in tubes according to the clinical laboratory guidelines for the laboratory test ordered. Blood for a complete blood count was collected in EDTA tubes, and blood for a basic metabolic panel or CRP was collected in sodium heparin tubes. These were the only clinical laboratory tests used to request remnant plasma for the study. The clinical lab stores samples at 4 degrees Celsius immediately after receipt, and the leftover blood from a clinical sample was requested by the study team. The clinical lab separated plasma from whole blood for samples collected in sodium heparin tubes, while the study team separated plasma from whole blood for samples collected in EDTA tubes by centrifugation (1,800 g × 5 min). We stored all remnant plasma samples with at least 250 microliters at −80°C until analysis.

### 2.3 Biomarker analysis

We selected subjects with at least two collected samples of adequate plasma volume (≥ 250 µl) for biomarker analysis. The volume of blood obtained from the patient was not altered for the purpose of this study. Therefore, samples with insufficient remnant plasma were excluded from the study. The multiplex assay measured a panel of inflammatory biomarkers: interleukin (IL)-6, IL-8, IL-10, IL-18, interferon gamma inducible protein (IP)-10, tumor necrosis factor alpha (TNF*α*), procalcitonin (PCT), human growth factor (HGF), endothelin growth factor (EGF), soluble suppression of tumorigenicity 2 (sST-2), and IL-1 receptor antigen (IL1-ra). We selected these biomarkers based on their role in the inflammatory response to bacterial infections and the existing literature (12, 20, 23, 24). Analyte concentrations were quantified using a customized multiplex Luminex® magnetic bead-based antibody assay (R&D Systems, Minneapolis, USA). Fluorescence signals for each biomarker bead region were analyzed on a Luminex®200, a dual-laser flow-based detection instrument. Concentrations below the lowest standard were recorded as the value of the lower limit of detection for statistical analyses. We used a 10-fold dilution to analyze PCT, sST-2, and IL-1ra, and the remaining biomarkers did not require dilution. Results of CRP measurement obtained for clinical use were obtained from the electronic health record. Clinical guidelines at the time of the study recommended measuring CRP at the time of suspected infection and when determining duration of treatment, but the decision to order a CRP was left to the discretion of the clinical team.

### 2.4 Clinical definitions

Samples were classified as “routine” if they were obtained from blood drawn for standard laboratory monitoring, usually a basic metabolic panel, and not within 48 h of suspected infection. We classified samples obtained at the time of a blood culture using the diagnosis of the event according to the following definitions:

A. Late-onset sepsis (LOS) (25): a positive blood culture obtained after 72 h of age and treated with at least five days of intravenous antibiotics. These were further categorized as sepsis due to Gram-positive (GP) or Gram-negative (GN) bacteremia according to the organism identified by blood culture.
B. Necrotizing enterocolitis (NEC) (26): radiographic evidence of necrotizing enterocolitis and clinical illness with or without a positive blood culture.
C. Clinical sepsis (CS): negative blood and urine cultures treated with ≥5 days of antibiotics for presumed infection due to clinical illness.
D. Sepsis Ruled Out (SRO): a negative blood culture treated with <5 days of empiric antibiotics while awaiting culture results and monitoring symptoms, typically for 48 h.

For the analysis, results of samples classified as SRO and routine were grouped as “no sepsis”.

### 2.5 Cardiorespiratory sepsis risk prediction

We previously developed a multivariable model to predict sepsis using continuous heart rate (HR) and oxygen saturation (SpO_2_) data called the Pulse Oximetry Warning Score, or POWS (17). The POWS model calculates the mean, standard deviation, skewness, kurtosis, and cross-correlation of HR and SpO_2_ every 10 min and uses logistic regression at each window to predict the relative risk of LOS with a positive blood culture in the next 24 h. We calculated hourly POWS values from continuous bedside monitoring data during the 12 h preceding blood cultures. POWS scores were calculated after discharge and thus did not influence decisions about clinical care. A heart rate characteristics (27, 28) monitoring system for sepsis risk prediction was in use during the study period, but was not routinely used in the decision to order a blood culture.

### 2.6 Statistical analyses

We compared distributions of inflammatory biomarkers and POWS data using Kruskal–Wallis analysis across the four diagnosis groups, followed by a pairwise Wilcoxon test with corrections for multiple testing. Hierarchical cluster analysis was performed on seven biomarkers in samples associated with a positive blood culture or NEC with no organism identified. Cytokine values were log transformed prior to clustering. The relationships between POWS and individual biomarker levels were assessed using univariate linear regression. The statistical significance of each model's coefficient was adjusted for repeated measures using the Huber-White method (29). A *p*-value <0.05 was considered statistically significant. The predictive performance of biomarkers and POWS were assessed individually or in combination using sensitivity, specificity at specific thresholds, and area under the receiver operator characteristics curve (AUC) using logistic regression to predict LOS or NEC. All statistical analysis was performed using RStudio (R Version 4.2.3, Vienna, Austria).

## 3 Results

### 3.1 Patients and samples

We enrolled 118 VLBW infants with parental consent. Of those enrolled, we had sufficient samples from 54 infants for analysis. The 54 infants included were 48% female, had a median gestational age of 26 weeks (IQR 25–27), and a median birth weight of 790 g (IQR 630–930). Compared with infants analyzed, infants excluded for insufficient samples or data were 43% female, had higher median gestational age (28 weeks, IQR 26–29) and birthweight (980 g, IQR 760–1,240). Table 1 shows clinical characteristics of infants included for sample analysis and compares those with and without LOS or NEC. In total, 188 plasma samples were analyzed, including 68 obtained near the time of blood cultures for suspected LOS or NEC and 120 at the time of routine blood sampling. The median number of samples analyzed per patient was 3 (range 2–6). Seven biomarkers (IP-10, IL-6, IL-10, IL-18, TNFa, IL-8, PCT) were analyzed at all times, while four (HGF, EGF, sST-2, IL-1ra) were successfully measured 80% of the time (152 samples in 45 patients).

**Table 1:** Cohort characteristics overall and grouped by infants with or with late-onset sepsis (LOS) or necrotizing enterocolitis (NEC) diagnosis.

	LOS/NEC (*N* = 30)	No LOS/NEC (*N* = 24)	Overall (*N* = 54)
Gestational age (weeks), mean (SD)	25.8 (2.1)	26.2 (1.6)	25.9 (1.9)
Birth weight (grams), mean (SD)	780 (203)	831 (245)	803 (222)
Male, *n* (%)	19 (63%)	9 (38%)	28 (52%)
Race/ethnicity, *n* (%)			
Black	3 (10%)	4 (17%)	7 (13%)
Hispanic	3 (10%)	1 (4%)	4 (7%)
White	23 (77%)	18 (75%)	41 (76%)
Unknown/other	1 (3%)	1 (4%)	2 (4%)
Died, *n* (%)	5 (17%)	0 (0%)	5 (9%)

Values are presented as mean (standard deviation) or number (%).

Of the 67 blood cultures with study samples analyzed, 28 were diagnosed as LOS or NEC with bacteremia. Of these, there were 22 cases of Gram-positive (GP) bacteremia, 5 Gram-negative (GN) bacteremia, and 1 with NEC and GN bacteremia. Organisms in the positive blood cultures were coagulase-negative Staphylococcus (CONS) species (*n* = 19), *Escherichia coli* (*n *= 2), *Enterobacter* species (*n *= 1), Group B *Streptococcus* (*n *= 1), *Klebsiella* species (*n *= 5), and methicillin-susceptible *Staphylococcus aureus* (*n *= 2). There were 2 cases of NEC with negative blood cultures and 17 negative blood cultures diagnosed as CS. The remaining 141 samples came from blood drawn at times with NS, including routine samples and blood cultures diagnosed as SRO.

### 3.2 Biomarkers

**Figure 1:**
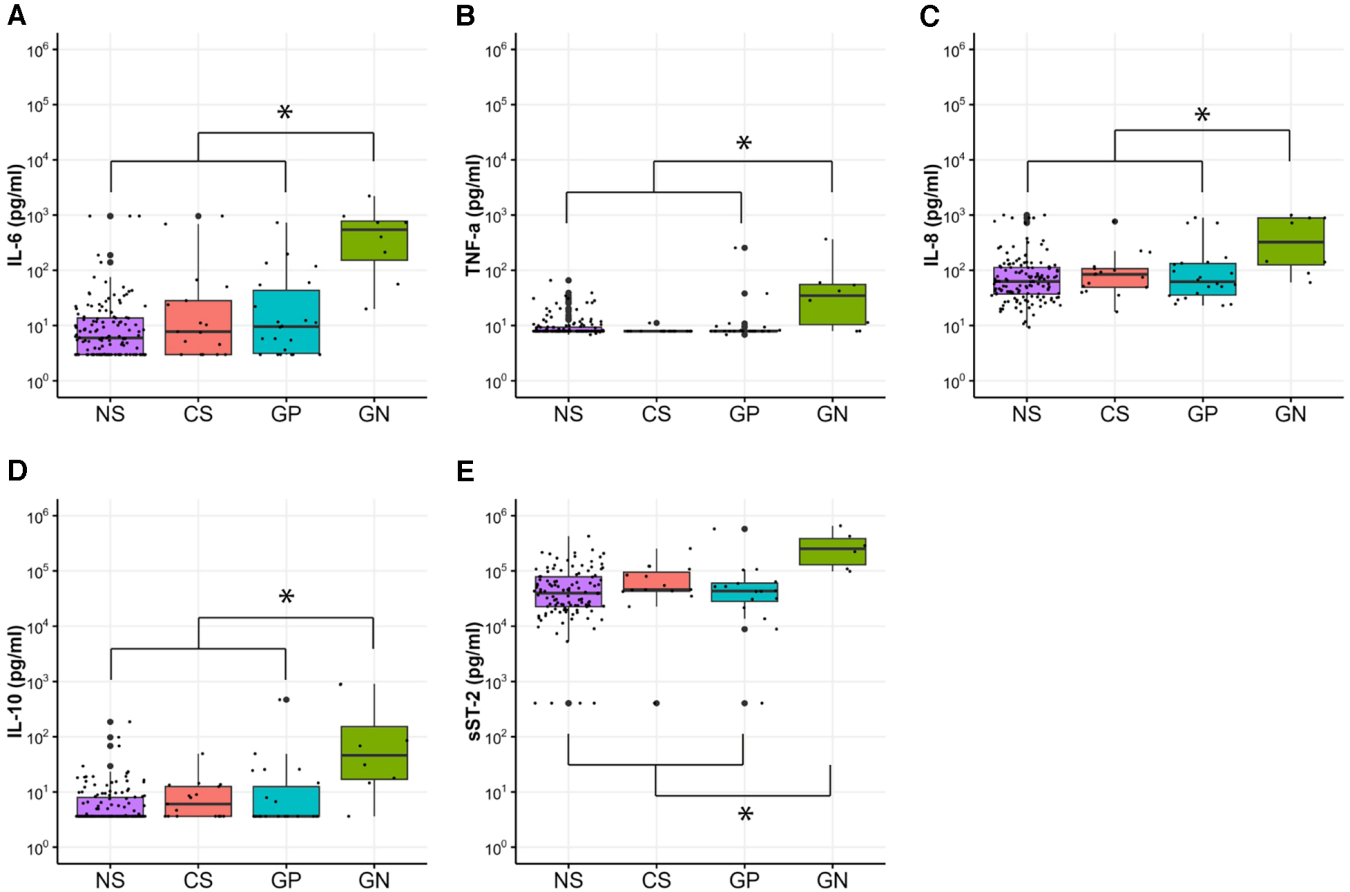
Plasma biomarker levels from remnant plasma collected at times with or without sepsis in very low birth weight (VLBW) infants. Samples were classified as clinical sepsis (CS, *n* = 17), Gram-positive bacteremia (GP, *n *= 22), Gram-negative bacteremia or Necrotizing Enterocolitis (NEC) (GN, *n* = 8), or no sepsis (NS, *n* = 141). IL-6 (**A**), TNF-a (**B**), IL-8 (**C**), IL-10 (**D**), and sST-2 (**E**) were significantly higher in patients with GN sepsis as compared with NS, CS, and GP sepsis, as indicated by asterisks. Biomarker levels were not different when comparing NS, CS, and GP sepsis samples.

Five of the eleven biomarkers (IL-6, TNF-α, IL-8, IL-10, and sST-2) were significantly higher in patients with GN sepsis or NEC than those with NS, CS, and GP sepsis (*p* < 0.05). Overall, biomarkers at CS or GP diagnosis were not significantly different from NS samples (Figure 1).

CRP was ordered by the clinical team at the time of 92 (49%) of the analyzed remnant plasma samples. CRP was significantly higher in patients with GN sepsis or NEC (median CRP 4.4 g/dl, IQR 2.1–11.0) compared to patients with no sepsis [median CRP 0.1 g/dl, IQR 0.1–0.4 (*p* < 0.05)] or clinical sepsis [median CRP 0.12 g/dl, IQR 0.1–1.2 (*p* < 0.05)]. There was no statistically significant difference in CRP for GN sepsis vs. GP sepsis cases [median CRP 2.7 g/dl, IQR 0.1–3.3 (*p* = 0.16)] or clinical sepsis [median CRP 0.12 g/dl, IQR 0.1–1.2 (*p* = 0.08)].

**Figure 2:**
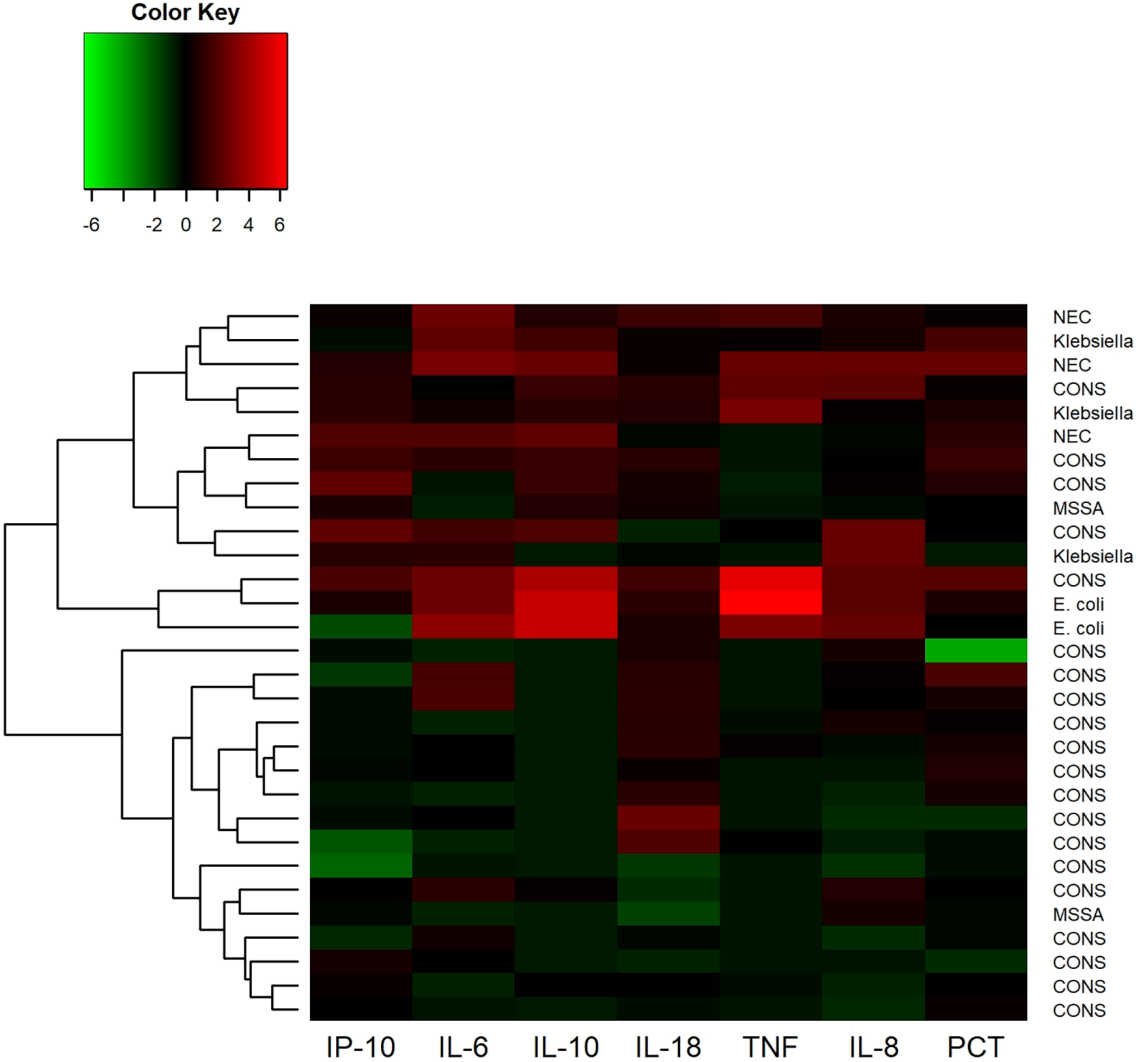
Hierarchical cluster analysis of cytokine levels in 30 cases of blood culture-positive late-onset sepsis (LOS) and necrotizing enterocolitis (NEC). The scaled natural log of each cytokine was taken prior to clustering. Higher than average cytokine levels are depicted in shades of red and lower than average levels are depicted in green. The resulting hierarchical clustering dendrogram is on the left-hand side of the heatmap.

Hierarchical cluster analysis of cytokines from 30 LOS or NEC blood samples showed two distinct clusters of biomarker profiles (Figure 2). GN bacteremia or NEC was more prevalent in the cluster of samples with the highest cytokine levels. IL-6 above 20 pg/ml had the best overall test accuracy for diagnosing gram-negative sepsis or NEC with a specificity of 78%, negative predictive value of 100%, and sensitivity of 100%.

### 3.3 Association with POWS, a cardiorespiratory sepsis risk prediction model

**Figure 3:**
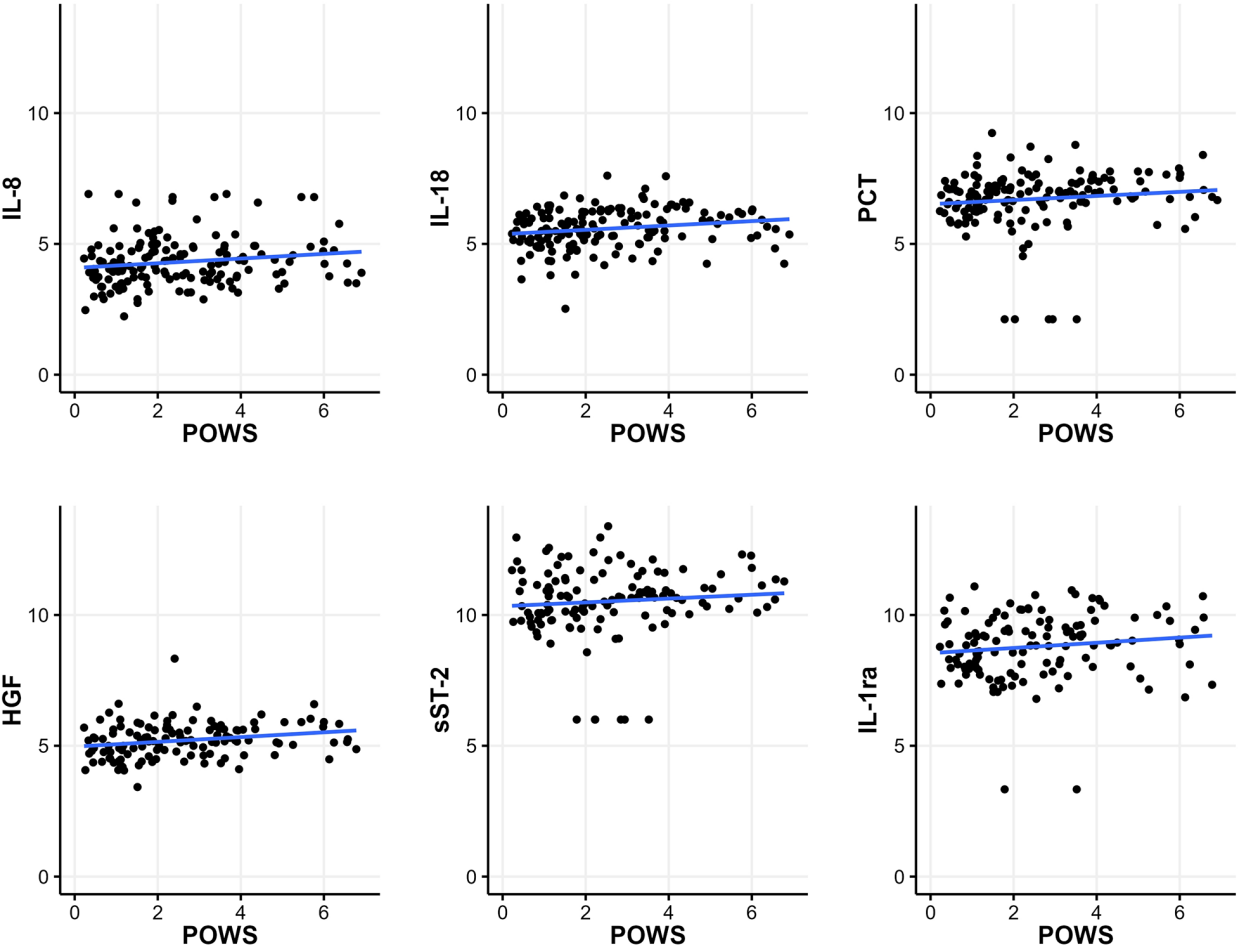
Relationship between the pulse oximetry warning score (POWS) and inflammatory biomarkers. Plots of the scaled natural log of each biomarker level on the y-axis and the maximum sepsis risk within 12 h before blood culture on the x-axis. POWS was significantly associated (*p* < 0.05) with the levels of the 5 biomarkers depicted in this figure (IL-8, PCT, HGF, sST-2, and IL1-ra), irrespective of the associated diagnosis.

Of the 188 samples, 187 had continuous heart rate and oxygen saturation data available around the time of sample collection to calculate POWS, a pulse oximetry warning score designed as a physiomarker for impending sepsis. POWS was significantly associated with the levels of 5 biomarkers (IL-8, PCT, HGF, sST-2, and IL1-ra) irrespective of the associated diagnosis (Figure 3, *p* < 0.05). The maximum POWS within the 12 h preceding the sample had an AUC to predict LOS (GN or GP) or NEC (with or without bacteremia) of 0.610. The AUC increased to 0.680 when the IL-6 level of the sample was added to the model.

## 4 Discussion

We assayed inflammatory biomarkers at the time of blood culture for suspected sepsis and routine laboratory testing in VLBW infants and found differences that distinguish late-onset Gram-negative sepsis and NEC from other diagnoses, but similar biomarker distributions obtained from samples collected at baseline and at the time of suspected sepsis or Gram-positive sepsis. We also found correlations between inflammatory biomarkers and POWS, an algorithm that detects abnormal patterns of heart rate and oxygen saturation that we previously developed as a physiomarker of sepsis (17).

Our results confirm the findings in a previous study of VLBW infants that multiple biomarkers can discriminate between GN sepsis and GP sepsis at the time of positive blood culture (12). Since Gram-negative infections and NEC are known to carry higher morbidity and mortality, this finding might be a useful adjunct clinical decision support tool in helping pick the appropriate empiric antibiotic therapy and institute treatment early (30–32). The current study adds to our prior work (12) because we analyzed samples remote from suspected sepsis. We found no significant difference in biomarker levels in these samples compared to those obtained at the time of clinical sepsis or Gram-positive sepsis. Some VLBW preterm infants may have a chronic or subacute systemic inflammatory response associated with lung disease or intracranial hemorrhage, which could confound sepsis prediction using hematologic biomarkers or cardiorespiratory physiomarkers (33–35).

Plasma cytokines and chemokines are early markers of immune activation in response to infection, and have been shown to rise in septic premature infants (22, 36, 37). Several studies have evaluated the utility of biomarkers for diagnosing sepsis by comparing biomarker levels in cases vs. controls (11). Others have compared cytokine profiles obtained at the time of suspected infection in VLBW infants when the blood culture returns positive vs. negative (38, 39). Instead, we assessed differences in inflammatory biomarkers measured at times when there was clinical suspicion for infection or sepsis and at times remote from infection. Kuster and colleagues also took the approach of measuring cytokines at baseline and at the time of suspected sepsis in VLBW infants and found IL-1ra and IL-6 to have high sensitivity and specificity in 21 cases of late-onset sepsis, even measured from samples taken the day prior to blood culture (37). A longitudinal study of extremely low birth weight (<1,000 g) measured cytokines at days 1, 3, 14, and 21 after birth and found that IFN-γ, IL-10, IL-18, TGF-β, and TNF-α levels differed among infants who developed fungal or bacterial LOS compared with those who never developed sepsis (22). This study enrolled a large, multicenter cohort but did not evaluate differences relative to the timing of blood cultures. They found that infants with higher levels of immune regulatory cytokines relative to pro-inflammatory cytokines were associated with an increased risk of LOS at any time during the NICU course (21).

Of the biomarkers assayed in our study, IL-6 has been most consistently identified as a promising sepsis biomarker in previous studies, though with variable sensitivity and specificity (39). We found that IL-6 had a high specificity at a threshold set to detect 100% of Gram-negative sepsis and NEC cases but low predictive accuracy for Gram-positive cases, which were mostly due to CONS bacteremia. IL-6 has been shown to predict fatal or severe sepsis in premature infants (40), which occurs more often in Gram-negative than Gram-positive infections due to pathogen virulence factors (41). However, we also found elevated levels of IL-6 measured from samples remote from sepsis with blood draws for routine laboratory tests.

The inclusion of sST-2 as a sepsis biomarker was novel for this population as its utility has mainly been studied in adult populations. It is an IL-1 family receptor that binds IL-33 and has been implicated in diseases involving intestinal inflammation (42). Recent studies in adult patients demonstrate an association between elevated sST-2 and the severity of illness in *Clostridium difficile* colitis (43, 44). Our results indicate that this may also be a useful biomarker for diagnosing NEC and Gram-negative sepsis, where gastrointestinal dysfunction and bacterial translocation cause systemic inflammation.

Cytokines and chemokines such as IL-6, IL-1ra, and IL-8 have been demonstrated to have diagnostic utility as early sepsis markers (37, 45), while acute phase reactants such as CRP and PCT rise during the later phases of systemic inflammation (46). CRP and PCT are also examples of the few inflammatory biomarkers available in U.S. clinical laboratories, which may drive their clinical use despite evidence of low clinical utility (14). A recent meta-analysis showed overall low sensitivity and specificity of CRP for LOS diagnosis (47).

We note several limitations of the study. First, the analysis was limited by the small sample size, where most late-onset sepsis events were due to CONS bacteremia, some of which may have represented contamination and not a true infection. Second, events diagnosed as clinical sepsis are heterogeneous in severity with subjective diagnostic criteria. We collected data on clinical characteristics, but not to the level of detail to account for concurrent inflammatory processes, such as lung disease, invasive mechanical ventilation, and minor procedures. Finally, the use of remnant plasma allowed us to enroll patients and collect samples without additional blood draws, but the volume of plasma available was small and therefore did not allow assays to be run in duplicate.

Analysis of plasma biomarkers and physiomarkers of LOS using POWS, a cardiorespiratory sepsis risk score, resulted in promising correlations that warrant further confirmation in larger studies. Continuous cardiorespiratory predictive monitoring used in conjunction with biomarker testing could prove useful, both for early initiation of antibiotics when the likelihood of sepsis is high and for sparing antibiotics when biomarkers and physiomarkers indicate low sepsis risk.

## 5 Conclusion

In conclusion, in a prospective cohort of VLBW infants, inflammatory biomarkers discriminated between late-onset sepsis due to gram-negative bacteremia or NEC and all other samples, including those collected at times with or without suspected sepsis. Biomarkers measured at the time of sepsis due to gram-positive bacteremia did not differ from those measured at times without sepsis. Several inflammatory biomarkers, measured at baseline and at the time of suspected or confirmed sepsis, correlated with cardiorespiratory physiomarkers of sepsis.

## Data Availability

The raw data supporting the conclusions of this article will be made available by the authors, without undue reservation.
